# Clinical Treatment Endpoints After Active Periodontal Treatment and 10 Years of Supportive Periodontal Care: A Retrospective Cohort Study

**DOI:** 10.1111/jcpe.14179

**Published:** 2025-07-08

**Authors:** Mario Schröder, Max Buchinger, Iulia Dahmer, Peter Eickholz, Hari Petsos

**Affiliations:** ^1^ Department of Periodontology Center for Dentistry and Oral Medicine (Carolinum) Goethe University Frankfurt Frankfurt/Main Germany; ^2^ Private Practice Germersheim Germany; ^3^ Private Practice Wiesbaden Germany; ^4^ Institute of Biostatistics and Mathematical Modeling Goethe University Frankfurt Frankfurt/Main Germany; ^5^ Center for Dentistry and Oral Medicine (Carolinum) Goethe University Frankfurt Frankfurt/Main Germany; ^6^ Private Practice Soest Germany

**Keywords:** endpoint, periodontal treatment, periodontitis, supportive periodontal care

## Abstract

**Objectives:**

Comparing periodontal stability following active periodontal treatment (APT/T1) and 120 ± 12 months of supportive periodontal care (SPC/T2) using four clinical endpoints (CEPs).

**Methods:**

CEP1: pocket probing depths (PPD) ≤ 4 mm, no sites with ≥ 4 mm with bleeding on probing (BOP), and total BOP < 10%; CEP2: no PPD > 4 mm with BOP or PPD ≥ 6 mm; CEP3: ≤ 4 sites with PPD ≥ 5 mm; CEP4: ≤ 5 teeth with PPD ≥ 5 mm. Assuming CEPs are mutually exclusive, patient‐ and tooth‐related parameters (e.g., periodontal tooth loss: PTL) were compared. Using receiver operating characteristic analysis for the prediction of PTL as a cutoff for CEP was assessed.

**Results:**

From 128 patients (age 65.5 ± 10.5 years; 83 stage III, 45 stage IV; 47 grade B, 81 grade C), 7 achieved CEP1, 23 CEP2, 45 CEP3, 23 CEP4, 30 noCEP at T1. At T2, six patients reached CEP1, 37 CEP2, 38 CEP3, 35 CEP4, 12 noCEP. For noCEP, the number of sites with PPD > 5 mm increased significantly, and PTL was higher compared to CEP1, CEP2 and CEP3 (*p* < 0.001).

**Conclusions:**

While achieving CEP1 is possible through comprehensive APT, treating a chronic disease often leads to less ideal CEP2/CEP3. Achieving CEP1, CEP2 or CEP3 after APT made no observable difference regarding PTL.

**Trial Registration:** The study is registered with the United States National Library of Medicine in the clinical trials database (URL: https://clinicaltrials.gov; NCT03048045)

## 1 Introduction

Clinical practice guidelines provide evidence‐based treatment recommendations for controlling periodontitis (Herrera et al. 2022; Sanz et al. 2020). However, treated patients remain at high risk of disease recurrence (Fardal and Linden 2005). Clinical treatment endpoints (CEPs) can be used to describe treatment results and are potentially effective tools to estimate the stability of periodontitis patients after comprehensive treatment. Well‐defined CEPs may also facilitate greater comparability between clinical studies and provide objective orientation for treatment results in clinical practice (Loos and Needleman 2020). True endpoints, such as tooth loss (TL), typically occur over long time periods (≥ 5 years), making them impractical. Tangible parameters accessible in daily practice are needed to judge clinical conditions in the short term. Thus, surrogate parameters narrowly associated with the future occurrence of true endpoints are applied; for example, deep periodontal pockets may predict future TL. The 2018 Classification of Periodontal and Peri‐implant Diseases and Conditions defines an ideal CEP of a successfully treated and stable periodontitis patient: a state of periodontal health with the absence of clinically detectable inflammation (Chapple et al. 2018; Papapanou et al. 2018). This CEP should ideally be achieved before and maintained during supportive periodontal care (SPC). However, the defined parameters (i.e., probing pocket depth (PPD) ≤ 4 mm, no site ≥ 4 mm with bleeding on probing [BOP]) and total BOP (< 10%: CEP1) may be unrealistic for many patients. This leads to the question of whether alternative, less strict CEPs may also predict periodontal stability for the majority of patients in the long term.

The European Federation of Periodontology (EFP) S3‐level clinical practice guideline defines another endpoint (CEP2) (Sanz et al. 2020). Another alternative is a treat‐to‐target approach (CEP3) (Feres et al. 2020). Since 2005, the Department of Periodontology of Goethe‐University Frankfurt has introduced a specific CEP, which defines disease recurrence when exceeded (CEP4). This approach defines a clinical threshold, above which the risk of periodontal deterioration is considered too high to be managed with SPC only (Petsos et al. 2020).

This study is part of an ongoing retrospective cohort study analysing 10‐year data from periodontal patients. This explorative analysis aimed to investigate periodontal stability measured primarily by TL due to periodontal reasons (PTL) after 10 years of SPC, and its association with four proposed CEPs immediately after active periodontal treatment (APT). Furthermore, we aimed to identify predictors influencing the attainment of different CEPs after APT.

## 2 Materials and Methods

This study analyses new and existing data obtained from a retrospective cohort study investigating long‐term outcomes of SPC. Protocols for clinical examinations and periodontal therapy have been described in detail previously (Petsos et al. 2020).

### 2.1 Patients

A combination of electronic and manual searches was conducted in the dental performance system to identify eligible study participants who had undergone APT at the Department of Periodontology of Goethe‐University Frankfurt/Main after October 2004 (private practice) or April 2005 (statutory insurance). Comprehensive periodontal treatment included oral hygiene instructions and supragingival debridement, followed by subgingival instrumentation (SI) based on a modified version (Eickholz et al. 2013) of the full‐mouth disinfection (FMD) concept (Quirynen et al. 1995). The protocol has been published in detail before (Eickholz et al. 2013; Petsos et al. 2020). A detailed description is provided as Supporting Information.

This retrospective cohort study was originally designed to include at least 100 patients reaching 120 ± 12 months after completion of APT (T1). Instead of stopping recruitment when 100 patients were included, we continued to recruit all patients who met the inclusion criteria to date. Both non‐surgical and surgical therapy had to be completed for T1. Before the study, all participants were informed in detail about the risks of participation and gave written informed consent at the 10‐year re‐examination (T2).

### 2.2 Supportive Periodontal Care

SPC was conducted in a university setting by dentists collaborating with dental nurses or dental hygienists, or by supervised undergraduate students. SPC was consistently administered throughout the entire follow‐up period, as long as the patient participated regularly. SPC has been reported in detail before (Petsos et al. 2021). A detailed description is provided as Supporting Information.

Data at T0 and T1 were extracted from patients' charts retrospectively (Petsos et al. 2020).

### 2.3 10 Year Re‐Examination (T2)

All qualifying patients were invited to participate in the T2 examination. Four different periodontists (K.N., T.R., P.E. and H.P.) were involved in re‐examinations and assessed the following parameters. The 10‐year examination has been reported before (Petsos et al. 2021). A detailed description is provided as Supporting Information.

### 2.4 Clinical CEPs

At T1 and T2, the number of patients who reached none or one of the following four CEPs was compared.

- **CEP1—Successfully treated stable periodontitis patient** (Chapple et al. 2018): PPD ≤ 4 mm (no site ≥ 4 mm with BOP) and total BOP < 10%.
- **CEP2—Endpoint of therapy** (Sanz et al. 2020): No PPD > 4 mm with BOP and no PPD ≥ 6 mm.
- **CEP 3—Treat‐to‐target approach** (Feres et al. 2020): ≤ 4 sites with PPD ≥ 5 mm irrespective of BOP.
- **CEP4—Frankfurt department‐specific definition for the requirement of systematic periodontal re‐treatment (recurrent case), when exceeded**: ≤ 5 teeth with PPD ≥ 5 mm irrespective of BOP.
- **noCEP—Patients who did not reach CEPs 1–4 according to the corresponding definitions above at T1 and/or T2 were included in this category**.

### 2.5 Statistical Analysis

Due to varying sources, there is some overlap in the defined CEPs. The group of patients classed as CEP2 encompasses CEP1, the group achieving CEP3 encompasses CEP1 and CEP2 and so forth. In contrast, the noCEP classification does not encompass any of the CEPs. This fact prevents comparison of CEPs within the same cohort. For purposes of comparison, CEPs were assumed to be mutually exclusive, and every patient was assigned a single CEP. In each case, the strictest applicable CEP was assigned to the patient (CEP1 > CEP2 > CEP3 > CEP4 > noCEP). CEP2 was considered stricter than CEP3. For CEP3, a single tooth exhibiting > 4 sites with PPD ≥ 5 mm is sufficient for exclusion from the classification. For CEP4, the presence of at least one site with PPD ≥ 5 mm on at least six different teeth is sufficient for exclusion. To account for overlapping CEPs, we quantified the number of teeth, TL and PTL for actual CEPs rather than the mutually exclusive classifications. The so‐called actual CEP encompasses all patients actually fulfilling the requirements of the respective CEP, for example, actual CEP3 encompasses CEP1 + CEP2 + CEP3. CEP1 and noCEP do not overlap with other CEPs. Investigation of potential predictors for the success of the CEPs was also performed for the actual CEPs and not for the mutually exclusive classifications.

Statistical analysis was performed using R (packages ‘RVAIdeMemoire’, ‘PMCMRplus’, ‘rcompanion’, ‘stats’, ‘coin’) and RStudio and included descriptive analysis of categorical (absolute and relative frequencies) and continuous (mean and standard deviation) variables for the whole sample, grouped by classification of CEP at T1 and T2. The distributions of the patients among the five groups (CEP1, CEP2, CEP3, CEP4 and noCEP) at T1 and T2 were analysed using the multinomial goodness‐to‐fit test. Comparisons between groups were performed for each time point using ANOVA (when the data were normally distributed) or the Kruskal–Wallis test (otherwise) for continuous data, and using the Fisher's exact test for categorical data. In the case of significant results, post hoc pairwise comparisons were conducted. The normality of the data was checked using the Shapiro–Wilk test. Comparisons between the frequencies of the patients achieving each CEP at T1 and T2 were performed using McNemar's test. Comparisons of long‐term stability parameters (BOP, number of teeth, PPD, CAL, number of sites with PPD > 5 mm and number of sites with PPD > 6 mm) between T1 and T2 were conducted using paired t‐tests when the data were normally distributed and the Wilcoxon matched pairs test otherwise.

Univariate and multiple logistic regressions were conducted for each time point (T1 and T2) and each of the actual CEPs separately (actual CEP2 = CEP2 + CEP1, actual CEP3 = CEP3 + CEP2 + CEP1, actual CEP4 = CEP4 + CEP3 + CEP2 + CEP1) in order to identify predictors for the achievement of the CEPs (Tables S1 and S2). A detailed description of this analysis is provided in the Supporting Information.

Finally, two logistic regressions were calculated, one to explain TL during SPC and another to explain PTL during SPC. The response variable in each case was the dichotomised version of the original variable (which recorded the number of lost teeth). The dichotomised variable recorded whether there were lost teeth (0 = no lost teeth, 1 = at least one tooth lost). CEP at T1, plaque (PCR), adherence (yes/no), repeated treatment (yes/no), smoking (yes/no) and diabetes (yes/no) were considered as predictors. In addition, ROC analysis was performed for comparing the predictive power of the CEPs achieved at T1 (the predictive variable was the ordinal variable equal to the number of not achieved actual CEPs) for long‐term stability encompassed by PTL_SPC (dichotomised variable).

## 3 Results

### 3.1 Patients and Patient‐Related Parameters

A total of 187 patient files were systematically reviewed to determine eligibility with the inclusion criteria (Figure S1). All 128 included subjects were diagnosed with periodontitis stage III or IV at baseline (T0; Table 1).

**TABLE 1:** Baseline (T0) patient characteristics grouped according to achievement of clinical endpoints (CEPs) at T1 (*p* < 0.05 in bold).

Parameter	Total	CEP1	CEP2	CEP3	CEP4	NoCEP	*p*
	(*n* = 128)	(*n* = 7; 5.5%)^A,B,C,D^	(*n* = 23; 18.0%)^A,E^	(*n* = 45; 35.2%)^B,E,F^	(*n* = 23; 18.0%)^C,F^	(*n* = 30; 23.4%)^D^	< 0.001***
Gender (male/female) (*n*/%)	65 (50.8%)/63 (49.2%)	3 (42.9%)/4 (57.1%)	14 (60.9%)/9 (39.1%)	21 (46.7%)/24 (53.3%)	9 (39.1%)/14 (60.9%)	18 (60%)/12 (40%)	0.457*
Age (mean ± SD)	54.3 ± 10.52	60.26 ± 8.5	54.9 ± 8.9	53.5 ± 11.3	55.3 ± 10.2	53 ± 11.2	0.797**
Smoking (*n*/%)							0.406*
Non‐smoker	104 (81.2%)	5 (71.4%)	19 (82.6%)	39 (86.7%)	20 (87%)	21 (70%)	
Former smoker	7 (5.5%)	1 (14.3%)	1 (4.4%)	3 (6.7%)	0 (0%)	2 (6.7%)	
Active smoker	17 (13.3%)	1 (14.3%)	3 (13%)	3 (6.7%)	3 (13%)	7 (23.3%)	
Diabetes (*n*/%)	5 (3.9%)	2 (28.6%)	0 (0%)	2 (4.4%)	1 (4.4%)	0 (0%)	**0.033** *
Stage (*n*/%)							0.628*
Stage III	83 (64.8%)	4 (57.1%)	14 (60.9%)	29 (64.4%)	18 (78.3%)	18 (60%)	
Stage IV	45 (35.2%)	3 (42.9%)	9 (39.1%)	16 (35.6%)	5 (21.7%)	12 (40%)	
Grade (*n*/%)							0.962*
Grade B	47 (36.7%)	3 (42.9%)	9 (39.1%)	15 (33.3%)	8 (34.8%)	12 (40%)	
Grade C	81 (63.3%)	4 (57.1%)	14 (60.9%)	30 (66.7%)	15 (65.2%)	18 (60%)	
Antibiotics (T1) (*n*/%)	19 (14.8%)	0 (0%)	0 (0%)^A^	9 (20%)	1 (4.4%)	9 (30%)^A^	**0.007** *
Surgery (T1) (*n*/%)	62 (48.4%)	2 (28.6%)	8 (34.8%)	22 (48.9%)	12 (52.2%)	18 (60%)	0.351*

*Note*: Superscript letters indicate significant pairwise comparisons.

Abbreviation: SD, standard deviation.

* Fisher exact test.

** ANOVA.

*** Goodness‐to‐fit multinomial test.

At T2, between June 2015 and July 2022, a total of 128 patients were re‐examined after 10.2 ± 0.5 years of SPC (Table 2). Between T1 and T2, 17 (13.3%) patients received repeated APT (Table 2). No repeated APT was conducted in CEP1 patients. Sixty‐eight (53.1%) patients were classified as adherent to SPC. Tables 1 and 2 provide patient characteristics at T0 and T1.

**TABLE 2:** Patient characteristics at T1 grouped according to achievement of clinical endpoints (CEPs) at T2 (*p* < 0.05 in bold).

Parameter	Total	CEP1	CEP2	CEP3	CEP4	NoCEP	*p*
	(*n* = 128)	(*n* = 6; 4.7%)^A,B,C^	(*n* = 37; 28.9%)^A,D^	(*n* = 38; 29.7%)^B,E^	(*n* = 35; 27.3%)^C,F^	(*n* = 12; 9.4%)^D,E,F^	< 0.001***
Adherent patients (*n*/%)	68	2 (33.3%)^A,B,C^	23 (62.2%)^A,D^	20 (52.6%)^B,E^	19 (54.3%)^C,F^	4 (33.3%)^D,E,F^	**< 0.001** ***
Gender (male/female) (*n*/%)	65 (50.8%)/63 (49.2%)	3 (50%)/3 (50%)	14 (37.8%)/23 (62.2%)	22 (57.9%)/16 (42.1%)	20 (57.1%)/15 (42.9%)	6 (50%)/6 (50%)	0.428*
Age (mean ± SD)	55.5 ± 10.5	44.4 ± 11.5	55.77 ± 9.53	58.7 ± 10.8	54.7 ± 10.3	51.8 ± 8.7	0.113**
Smoking (*n*/%)							0.296*
Non‐smoker	104 (81.2%)	6 (100%)	32 (86.5%)	32 (85.2%)	27 (77.1%)	7 (58.3%)	
Former smoker	7 (5.5%)	0 (0%)	3 (8.1%)	1 (2.6%)	2 (5.7%)	1 (8.3%)	
Active smoker	17 (13.3%)	0 (0%)	2 (5.4%)	5 (13.2%)	6 (17.1%)	4 (33.3%)	
Diabetes (*n*/%)	5 (3.9%)	0 (0%)	1 (2.7%)	2 (5.3%)	2 (5.7%)	0 (0%)	0.938*
Stage (T0) (*n*/%)							0.607*
Stage III	83 (64.8%)	2 (33.3%)	24 (64.9%)	26 (68.4%)	23 (65.7%)	8 (66.7%)	
Stage IV	45 (35.2%)	4 (66.7%)	13 (35.1%)	12 (31.6%)	12 (34.2%)	4 (33.3%)	
Grade (T0) (*n*/%)							0.161*
Grade B	47 (36.7%)	4 (66.7%)	18 (48.7%)	12 (31.6%)	10 (28.6%)	3 (25%)	
Grade C	81 (63.3%)	2 (33.3%)	19 (51.4%)	26 (68.4%)	25 (71.4%)	9 (75%)	
Recurrence (T2) (*n*/%)	17 (13.3%)	0 (0.%)	5 (13.5%)	3 (7.9%)	5 (14.3%)	4 (33.3%)	0.245*
Antibiotics (T1) (*n*/%)	19 (14.8%)	0 (0%)	5 (13.5%)	6 (15.8%)	5 (14.3%)	3 (25%)	0.798*
Surgery (T1) (*n*/%)	62 (48.4%)	1 (16.7%)	17 (46%)	17 (44.7%)	22 (62.9%)	5 (41.7%)	0.224*

*Note*: Superscript letters indicate significant pairwise comparisons.

Abbreviation: SD, standard deviation.

* Fisher exact test.

** ANOVA.

*** Goodness‐to‐fit multinomial test.

### 3.2 Tooth‐Related Parameters

From T0 (*n* = 3126, 24.4 ± 3.7 teeth/patient) the number of teeth decreased at T1 (*n* = 3080, 24.1 ± 3.9 teeth/patient) and decreased further until T2 (2916 teeth, 22.8 ± 4.8 teeth/patient). Consequently, 164 teeth were lost during SPC (1.3 ± 1.8 teeth/patient) of which 55 (34%, 0.4 ± 0.9 teeth/patient) were lost due to periodontal reasons. Tables 3 and 4 show tooth‐related parameters.

**TABLE 3:** Tooth‐related baseline (T0) characteristics grouped according to achievement of clinical endpoints (CEPs) at T1 (*p* < 0.05 in bold).

Parameter	Total	CEP1	CEP2	CEP3	CEP4	NoCEP	*p*
	(*n* = 128)	(*n* = 7)	(*n* = 23)	(*n* = 45)	(*n* = 23)	(*n* = 30)	
Teeth (mean ± SD)	24.4 ± 3.7	21.1 ± 3.1^A^	25.3 ± 3.2^A^	24.9 ± 2.8	24.1 ± 4.8	24 ± 4.3	**0.045** *
PPD (mean ± SD)	3.3 ± 0.7	2.9 ± 0.5^A^	3 ± 0.5^B^	3.3 ± 0.7^C^	3.2 ± 0.5	3.6 ± 0.7^A,B,C^	**0.001** *
Number of sites with PPD > 5 mm (mean ± SD)	26.6 ± 19.7	12.1 ± 13.3^A^	18.7 ± 14.5^B^	26.3 ± 21.9^C^	23.8 ± 13.2	38.8 ± 19.4^A,B,C^	**< 0.001** *
Number of sites with PPD > 6 mm (mean ± SD)	15.6 ± 14.8	4.4 ± 4.7^A^	9.2 ± 7.8^B^	15.6 ± 17.4^C^	15.4 ± 11.9	23.3 ± 15.1^A,B,C^	**< 0.001** *
CAL (mean ± SD)	3.8 ± 0.9	3.7 ± 1	3.5 ± 0.8	3.8 ± 1	3.7 ± 0.7	4.1 ± 1.1	0.183*
PISA (mean ± SD)	413 ± 381	105 ± 112^A^	375 ± 422	374 ± 344	389 ± 402	592 ± 368^A^	**0.006** *
Mobile teeth (class II) per patient (mean ± SD)	1.5 ± 2	0.6 ± 1.1	1.6 ± 2.7	1.4 ± 2	1.4 ± 1.6	1.7 ± 2	0.483*
Mobile teeth (class III) per patient (mean ± SD)	0.4 ± 0.8	0.3 ± 0.5	0.2 ± 0.5	0.4 ± 0.9	0.6 ± 1	0.5 ± 0.6	0.293*
Furcation involved teeth (class II) per patient (mean ± SD)	1.6 ± 1.7	1.7 ± 1	1.4 ± 1.9	1.6 ± 1.6	1.9 ± 1.4	1.7 ± 2	0.508*
Furcation involved teeth (class III) per patient (mean ± SD)	0.7 ± 1.1	0.6 ± 1.5	0.6 ± 1.1	0.5 ± 1	0.9 ± 1.1	1 ± 1.1	0.096*

*Note*: Superscript letters indicate significant pairwise comparisons.

Abbreviations: APT, active periodontal treatment; CAL, clinical attachment level; PISA, periodontal inflamed surface area; PPD, periodontal probing depth; PTL, periodontal tooth loss; SD, standard deviation; TL, tooth loss.

* Kruskal–Wallis test.

**TABLE 4:** Tooth‐related characteristics (T1) grouped according to achievement of clinical endpoints (CEPs) at T2 (*p* < 0.05 in bold).

Parameter	Total	CEP1	CEP2	CEP3	CEP4	NoCEP	*p*
	(*n* = 128)	(*n* = 6)	(*n* = 37)	(*n* = 38)	(*n* = 35)	(*n* = 12)	
Teeth (mean ± SD)	24.1 ± 3.9	26.7 ± 0.8	23.7 ± 4.6	24.1 ± 3.3	23.6 ± 4.1	25.3 ± 3.2	0.241*
PPD (mean ± SD)	2.5 ± 0.3	2.3 ± 0.3	2.5 ± 0.4	2.5 ± 0.3	2.6 ± 0.4	2.6 ± 0.4	0.489**
Number of sites with PPD > 5 mm (mean ± SD)	5.8 ± 5.5	1.5 ± 2.1^A,B^	5.1 ± 5.7	4.9 ± 4.9	7.8 ± 6^B^	7.2 ± 4.3^A^	**0.005** *
Number of sites with PPD > 6 mm (mean ± SD)	2.2 ± 2.7	0.3 ± 0.8^A^	2.1 ± 2.5	1.3 ± 1.8^B^	3.5 ± 3.6^A,B^	2.6 ± 2.2	**0.002** *
CAL (mean ± SD)	3.2 ± 0.8	2.5 ± 0.4	3.3 ± 0.9	3.2 ± 0.7	3.4 ± 0.8	3.1 ± 0.8	0.09
PISA (mean ± SD)	189 ± 152	99 ± 52	164 ± 130	190 ± 132	200 ± 145	278 ± 266	0.372*
Mobile teeth (class II) per patient (mean ± SD)	0.6 ± 1.2	0 ± 0	0.5 ± 1.1	0.5 ± 1.2	0.9 ± 1.4	0.3 ± 0.5	0.22*
Mobile teeth (class III) per patient (mean ± SD)	0.2 ± 0.4	0 ± 0	0.1 ± 0.4	0.2 ± 0.4	0.3 ± 0.4	0.2 ± 0.4	0.508*
Furcation involved teeth (class II) per patient (mean ± SD)	1 ± 1.6	0.8 ± 1.2	1.6 ± 2	0.6 ± 0.7	0.8 ± 1.3	0.8 ± 2.3	0.121*
Furcation involved teeth (class III) per patient (mean ± SD)	0.5 ± 1	1.2 ± 0.4	0.4 ± 0.8	0.3 ± 0.8	0.7 ± 1	0.5 ± 1.5	0.07*

*Note*: Superscript letters indicate significant pairwise comparisons.

Abbreviations: CAL, clinical attachment level; PISA, periodontal inflamed surface area; PPD, periodontal probing depth; PTL, periodontal tooth loss; SD, standard deviation; SPC, supportive periodontal care; TL, tooth loss.

* Kruskal–Wallis test.

** ANOVA.

### 3.3 Comparison of CEPs

The distribution of CEPs at T1 is provided in Table 1, and their distribution at T2 in Table 2. Between T1 and T2, the distribution of CEPs was significantly different (*p* < 0.001) (Table 5).

**TABLE 5:** Frequencies of the clinical endpoints (CEPs) achieved at T1 and T2 and CEP changes between T1 and T2 depending on T1 CEP achievement, that is, how many individuals switched from one CEP to another or maintained one specific CEP (*p* < 0.05 in bold).

	T2 CEP1 (*n* = 6)			T2 CEP2 (*n* = 37)		T2 CEP3 (*n* = 38)		T2 CEP4 (*n* = 35)		T2 noCEP (*n* = 12)		*p* *
		*n*	%	*n*	%	*n*	%	*n*	%	*n*	%	
T1 CEP1 (*n* = 7)	*n*	1	14.3%	1	14.3%	3	42.9%	2	28.6%	0	0%	0.624
	%	16.7%		2.7%		7.9%		5.7%		0%		
T1 CEP2 (*n* = 23)	*n*	2	8.7%	9	39.1%	7	30.4%	4	17.4%	1	4.3%^C^	**0.042**
	%	33.3%		24.3%		18.4%		11.4%		8.3%		
T1 CEP3 (*n* = 45)	*n*	3	6.7%^A,B^	14	31.1%^A,C^	16	35.6%^B,D^	9	20.0%^C^	3	6.7%^C,D^	**0.002**
	%	50%		37.8%		42.1%		25.7%		25.0%		
T1 CEP4 (*n* = 23)	*n*	0	0%^A^	6	26.1%	5	21.7%	10	43.5%^A^	2	8.7%	**0.006**
	%	0%		16.2%		13.2%		28.6%		16.7%		
T1 noCEP (*n* = 30)	*n*	0	0%^A^	7	23.3%	7	23.3%	10	33.3%^A^	6	20%	**0.02**
	%	0%		18.9%		18.4%		28.6%		50%		

*Note*: Superscript letters indicate significant pairwise comparisons.

* Goodness‐to‐fit multinomial test.

### 3.4 Predictors for the Achievement of the Actual CEPs

Regression analyses revealed a significant (*p* = 0.027) inverse relationship between baseline (T0) number of teeth and CEP1 achievement. The number of sites with PPD > 6 mm at T0 was identified as a predictor for not achieving actual CEP2 at T1 (*p* = 0.002). Additionally, the number of sites with PPD > 5 mm at T0 was identified as a predictor for not achieving actual CEP3 (*p* = 0.03) or actual CEP4 (*p* < 0.001) (Table 6a).

**TABLE 6a:** Associations between predictors at T0 and achieved actual clinical endpoints (CEPs) at T1 (*p* < 0.05 in bold).

Clinical endpoints (T1)	Predictor (T0)	*p*	Regression coefficient	Odds ratio with confidence interval
CEP1	Diabetes	0.057	2.12	8.37 (0.828, 79.38)
	Number of teeth	**0.027**	−0.2	0.82 (0.68, 0.98)
	PISA	0.083	−0.005	0.994 (0.987, 0.999)
Actual CEP2 (CEP1 + CEP2)	Number of sites with PPD > 6 mm	**0.002**	−0.07	0.93 (0.88, 0.97)
Actual CEP3 (CEP1 + CEP2 + CEP3)	Number of sites with PPD > 5 mm	**0.03**	−0.02	0.98 (0.96, 1)
	Mobility (0, I/II, III)	0.11	−0.66	0.52 (0.23, 0.14)
Actual CEP4 (CEP1 + CEP2 + CEP3 + CEP4)	Number of sites with PPD > 5 mm	**< 0.001**	−0.03988	0.96 (0.94, 0.98)

There was a significant negative association between age at T1 and achievement of CEP1 at T2 (*p* = 0.038). Grade C was associated with lower chances of achieving actual CEP2 (*p* = 0.017). Additionally, a significant association was shown between actual CEP4 achievement and T1 number of sites with PPD > 6 mm (*p* = 0.002). Smoking status at T1 was identified as a predictor for not achieving actual CEP4 and thus any CEP (*p* = 0.034) at T2 (Table 6b). The additional sensitivity analyses revealed that the results of the original analysis were only partially replicable with this model (Tables S3 and S4). The significant association between actual CEP4 achievement at T1 and T0 number of sites with PPD > 5 mm was confirmed (*p* < 0.001) (Table S3). Both analyses indicate that an increase in the number of sites with PPD > 5 mm decreases the chances of achieving a better CEP. Also, the significant negative association of smoking at T1 with achieving actual CEP2 and actual CEP4 at T2 was confirmed (*p* = 0.007) (Table S4). This indicates that our results should be interpreted with caution.

**TABLE 6b:** Associations between predictors at T1 and achieved actual clinical endpoints (CEPs) at T2 (*p* < 0.05 in bold).

Clinical endpoints (T2)	Predictor (T1)	*p*	Regression coefficient	Odds ratio with confidence interval
CEP1	Age	**0.038**	−0.11	0.9 (0.81, 0.99)
	CAL	0.06	−1.72	0.18 (0.02, 0,89)
Actual CEP2 (CEP1 + CEP2)	Grade C	**0.017**	−0.92	0.38 (0.18, 0.85)
Actual CEP3 (CEP1 + CEP2 + CEP3)	Smoking	0.06	−0.51	0.60 (0.35, 1.02)
	Number of sites with PPD > 6 mm	**0.002**	−0.23	0.79 (0.68, 0.91)
Actual CEP4 (CEP1 + CEP2 + CEP3 + CEP4)	Smoking	**0.034**	−0.72	0.48 (0.25, 0.98)

*Note*: Statistic: multiple logistic regression.

Abbreviations: CAL, clinical attachment level; PISA, periodontal inflamed surface area; PPD, periodontal probing depth.

### 3.5 Long‐Term (T2) Stability According to CEP Achievement After Active Periodontal Treatment (T1)

Comparing mean PPD change from T1 to T2 in terms of CEP at T1, analysis failed to detect significant differences. For the change in number of sites with PPD > 5 mm, CEP1, CEP2 (*p* < 0.001) and CEP3 (*p* = 0.02) patients exhibited significant reduction compared to noCEP. CEP4 showed a small decrease, but significantly less compared to CEP2 (*p* < 0.04). For the change in the number of sites with PPD > 6 mm, CEP2 patients exhibited a significant reduction compared to noCEP (*p* = 0.03). CEP4 and noCEP patients showed an increase (Table 7a). The same changes were observed for PTL, which was significantly higher in noCEP compared to CEP1, CEP2 and CEP3 (*p* < 0.001) (Table 7a). At T1 CEP1 was a significant predictor for no TL (*p* < 0.042) (Table 7b), CEP2 (*p* = 0.012) and CEP3 (*p* = 0.002) significant predictors for no PTL during SPC (Table 7c). No significant differences regarding number of sites with PPD > 5 mm and PTL were revealed between CEP1, 2 and 3 (Table 7a). The results of the sensitivity and specificity analyses are presented in Table 5 and the ROC curve is shown in Figure S2. The best cutoff according to Youden's index is obtained for the decision rule ‘periodontal tooth loss during SPC (PTL_SPC) is to be expected if at most CEP4 is reached at T1’, that is, not achieving at least actual CEP3 at T1 is associated with PTL during SPC.

**TABLE 7a:** Changes in clinical parameters (T1‐T2) distributed among the endpoints achieved at T1.

	CEP1	CEP2	CEP3	CEP4	NoCEP	*p* *
	(*n* = 7)	(*n* = 23)	(*n* = 45)	(*n* = 23)	(*n* = 30)	
PPD (mean ± SD; mm)	−0.1 ± 0.3	0 ± 0.3	0 ± 0.3	0.1 ± 0.3	0.2 ± 0.5	0.245
Number of sites with PPD > 5 mm (mean ± SD; *n*)	−2.7 ± 2.4	−2.7 ± 2.4	−2.2 ± 4.7	−0.7 ± 4.2	3.5 ± 13.2	< 0.001 Post hoc: NoCEP‐CEP1: *p* < 0.001 NoCEP‐CEP2: *p* < 0.001 NoCEP‐CEP3: *p* = 0.02 CEP4‐CEP2: *p* = 0.04
Number of sites with PPD > 6 mm (mean ± SD; *n*)	−1.3 ± 1.7	−1.1 ± 2.3	−0.2 ± 2	1.2 ± 3.8	0.4 ± 6.4	0.03 Post hoc: NoCEP‐CEP2: *p* = 0.03
CAL (mean ± SD; mm)	−0.2 ± 0.3	0 ± 0.5	0 ± 0.6	−0.1 ± 0.3	0.1 ± 0.6	0.99
PISA (mean ± SD; mm^2^)	−123 ± 123	−118 ± 200	−42 ± 221	1 ± 156	−6 ± 241	0.25
BOP (mean ± SD; %)	−10.1 ± 10.9	−6.6 ± 11.6	−3 ± 14.7	−2.2 ± 8.5	−2 ± 13	0.61
TL during SPC (mean ± SD; *n*)	1.3 ± 2.3	0.9 ± 1.4	0.9 ± 1.4	1.6 ± 2.2	1.9 ± 2	0.12
PTL during SPC (mean ± SD; *n*)	0 ± 0	0.2 ± 0.7	0.2 ± 0.5	0.5 ± 0.8	1 ± 1.4	< 0.001 Post hoc: NoCEP‐CEP1: *p* < 0.001 NoCEP‐CEP2: *p* < 0.001 NoCEP‐CEP3: *p* < 0.001
		Actual CEP2 (CEP1 + 2) (*n* = 30)	Actual CEP3 (CEP1 + 2 + 3) (*n* = 75)	Actual CEP4 (CEP1 + 2 + 3 + 4) (*n* = 98)		
TL during SPC (mean ± SD; *n*)		1.0 ± 1.6	0.9 ± 1.5	1.1 ± 1.7		
PTL during SPC (mean ± SD; *n*)		0.2 ± 0.6	0.2 ± 0.5	0.3 ± 0.6		

*Note*: Superscript letters indicate significant pairwise comparisons.

Abbreviations: CAL, clinical attachment level; EP, endpoint; PISA, periodontal inflamed surface area; PPD, periodontal probing depth; PTL, periodontal tooth loss; SD, standard deviation; TL, tooth loss.

* Independence test based on ranks with stratifying variables ‘smoking’ and ‘diabetes’ and pairwise post hoc comparisons.

**TABLE 7b:** *p*‐values and odds ratios (OR) for tooth loss (TL_SPC) and for the CEPs in comparison with NoCEP at T1 (*p* < 0.05 and respective OR in bold).

	CEP1	CEP2	CEP3	CEP4
*p*	**0.042**	0.057	0.087	0.44
OR with 95% CI	**0.12 (0.01, 0.81)**	0.31 (0.09, 1.01)	0.41 (0.15, 1.12)	0.64 (0.2, 2.02)

**TABLE 7c:** *p*‐values and odds ratios (OR) for periodontal tooth loss (PTL_SPC) for the CEPs in comparison with NoCEP at T1 (*p* < 0.05 and respective OR in bold).

	CEP1	CEP2	CEP3	CEP4
*p*	0.989	**0.012**	**0.002**	0.121
OR with 95% CI	8.17 × 10^−9^ (NA, 5.6 × 10^40^)	**0.16 (0.03, 0.61)**	**0.16 (0.05, 0.49)**	0.39 (0.2, 1.25)

## 4 Discussion

CEPs are tools for assessing the efficacy of periodontal therapy and clinically evaluating treatment success on a patient level. Recently proposed CEPs reflect disease remission or control at the individual patient level (Chapple et al. 2018; Feres et al. 2020; Sanz et al. 2020). Interestingly, these CEPs each include different exclusively clinical thresholds of PPD, and most also assess BOP. They do not consider systemic factors, such as smoking or diabetes mellitus. However, few studies have investigated the extent to which these CEPs are achieved after APT and long‐term SPC (Bertl et al. 2022; Rattu et al. 2023), or their impact on long‐term periodontal health. It is currently debated whether these CEPs represent realistically achievable treatment outcomes for the majority of patients undergoing periodontal therapy (Feres et al. 2020; Bertl et al. 2022). Moreover, the practical relevance, applicability and long‐term stability of the proposed CEPs remain unclear (Hujoel 2004).

The findings of this retrospective cohort study indicate that few patients were able to achieve a state of stable periodontitis after APT (CEP1; 5.5%) (Chapple et al. 2018). A higher proportion of patients achieved the less rigorous CEP2 (actual CEP2 23.4%) (Sanz et al. 2020) and the treat‐to‐target CEP (actual CEP3; 58.6%) (Feres et al. 2020). Clinical stability after treatment of periodontitis can be affected by many factors, both patient‐related (e.g., smoking, adherence to SPC, individual oral hygiene, diabetes) (Eickholz et al. 2008; Chambrone and Chambrone 2006; Petsos et al. 2020) and tooth‐related (e.g., tooth type, furcation involvement, residual pockets) (Faggion et al. 2007; Petsos et al. 2021; Müller et al. 2013). However, it is unclear to what extent each of these factors also contributes to the difficulty in achieving CEPs immediately after APT. Even short‐term healing may be impaired by the general host response and factors such as individual oral hygiene (Nyman et al. 1977) and smoking (Cortellini et al. 2001). While issues of individual oral hygiene and smoking may be controlled (Sanz et al. 2020), the general host response cannot. Therefore, the chronic character of the disease may also partially explain the difficulty in achieving an ideal CEP (CEP1) in the short term (T1). However, its effect on long‐term results (T2) is much more significant.

In this retrospective analysis, we observed no significant differences in the changes of clinical parameters from T1 to T2 between patients who had achieved CEP1, CEP2 or CEP3 at T1 (Table 7a). In contrast, noCEP patients exhibited a significant increase in the number of sites with PPD > 5 mm compared to CEP1, CEP2 and CEP3, as well as a significant increase in sites with PPD > 6 mm compared to CEP2. For patients achieving CEP1, CEP2 and CEP3 at T1, PTL between T1 and T2 was significantly lower than in noCEP. There were no significant differences in PTL from T1 to T2 between CEP1, CEP2, CEP3 and CEP4 (Table 7a). When correcting for the confounding factors plaque, adherence, repeated treatment, smoking and diabetes, CEP2 and CEP3 at T1 were the only predictors of no PTL between T1 and T2 (Table 7c). Thus, these retrospective analyses do not suggest a benefit of a strictly defined state of stable periodontitis after APT (CEP1) compared to the less rigorous CEP2 (Sanz et al. 2020), or CEP3 (Feres et al. 2020) regarding long‐term clinical outcomes. This observation is confirmed by sensitivity testing. However, the only individuals who did not require repeated APT were those who achieved CEP1 at T1. In contrast, five individuals achieving CEP2 at T1 (17%), three with CEP3 (eight/11% with actual CEP3), five with CEP4 (13/13% with actual CEP4) and four classed as noCEP (13%) at T1 required repeated APT during SPC. This suggests that maintaining stability from CEP1 may require less effort (indicated by repeated APT) than less rigorous CEPs.

A recent meta‐analysis evaluated the prevalence of different CEPs, including CEP1 (Chapple et al. 2018), CEP2 (Sanz et al. 2020) and CEP3, (Feres et al. 2020), among others (Rattu et al. 2023). In our study, to enable comparison, each patient was assigned a single CEP, whereas the aforementioned meta‐analysis evaluated the respective prevalence of each CEP across the entire population. Achievement of CEP1 after APT or SPC was rare in our cohort (5.5% and 4.7%). Rattu et al. (2023) reported similar numbers, with only 9.4% of treated patients achieving CEP1 following APT according to the EFP S3‐level clinical practice guidelines (Herrera et al. 2022; Sanz et al. 2020). In a recent study conducted at the University of Vienna (Austria), approximately every fifth patient achieved CEP1 (Bertl et al. 2022). Patients enrolled in our study were treated in a university setting by board‐certified periodontists, postgraduates and supervised dental students (for SI/SPC only), which may explain differences regarding achieved CEPs between studies. In terms of CEP3, and in a sub‐analysis of studies with APT, approximately half of the cohort (52.4%) attained CEP3 after APT (Rattu et al. 2023). This is in contrast with our findings of 29.7% CEP3 at T1. These differences are likely explained by the fact that we assigned a single CEP for each patient, compared to the evaluation of the overall respective prevalence of each CEP (Rattu et al. 2023). The CEPs overlap: The cohort of patients achieving CEP2 encompasses CEP1, the cohort achieving CEP3 encompasses CEP1 and 2 and so forth. Thus, for purposes of comparison, we assumed CEPs as mutually exclusive. The sum of CEP1, CEP2 and CEP3 (actual CEP3: 58.6% at T1, 63.3% at T2) is comparable to the results reported by Rattu et al. (2023). The prevalence values for CEP3 are no coincidence. The treat‐to‐target concept (Feres et al. 2020) established a threshold for prospectively determining periodontal stability after APT, designed to include approximately half of the population (Feres et al. 2020). In general, achieving periodontal stability appears difficult, with only every second patient reaching a state of controlled periodontitis, according to both CEP2 and CEP3. Although the distribution of patients among the different CEPs showed only minor variations between T1 and T2, we observed a transition of patients from CEPs with more stringent criteria to those with looser criteria, and vice versa (Table 5).

The finding that only one patient was able to maintain CEP1 questions the relevance of ‘stable periodontitis’; CEP2, CEP3 and CEP4 appear to be more feasible for patients and therefore relevant to clinical practice. Few studies have investigated the maintenance or alteration of achieved CEPs during SPC. Bertl et al. (2022) demonstrated that CEP1 could not be maintained in all cases during SPC following APT. After a mean follow‐up period of 10.7 years, approximately 80% (17 of an initial 21 patients) maintained CEP1. Feres et al. (2020), examining CEP3, found that 88% of patients who attained CEP3 after APT were able to maintain it for two years. These results exceed those obtained in our study, given the much shorter observation period.

Bertl et al. (2022) identified missing CEP1 as a significant predictor of more diseased teeth at the last recorded SPC assessment and an increased risk of PTL. Regression analysis of our data identified several predictors at T1 for the likelihood of achieving CEPs at T2. The actual analysis found a significant inverse relationship between baseline number of teeth and CEP1 achievement, indicating that patients with fewer teeth show an increased probability of achieving CEP1. Fewer teeth meaning fewer sites at risk of unstable periodontitis (persisting PPD = 4 mm plus BOP or PPD > 4 mm) may explain this observation. Patients' age was a further factor affecting achievement of CEP1 and CEP3. Older age at baseline has been previously identified as a patient‐related risk factor for TL in this cohort (Petsos et al. 2020). Older patients may not be able to maintain effective oral hygiene due to limited motor function. Other factors, such as treatment expenses, limited mobility or lack of understanding of complex treatment plans, may contribute to lower adherence among older patients. Smoking status at T1 was also identified as a negative predictor for the achievement of any investigated CEP at T2. Smoking is known as a modifying risk factor in the development of periodontal disease (Tonetti et al. 2018).

There are several implications of attaining or failing to attain these CEPs. Considering the long‐term clinical stability, while most probing parameters remained constant over 10 years in CEP1, CEP2 and CEP3 patients, no significant differences were observed regarding quantitative changes in clinical parameters between the CEP1, CEP2 and CEP3 groups. This implies that achieving CEP2 or CEP3 (more feasible for most patients than CEP1) may be sufficient in predicting long‐term periodontal stability for the majority of patients. Significantly more tooth loss as the true end point was determined within noCEP between T1 and T2 than for CEP1: 0 teeth, CEP2: 0.22 teeth, CEP3: 0.2 teeth (*p* < 0.001) (Table 7a). In fact, the best cutoff rule for the prediction of PTL is not achieving at least CEP3 (Table 5).

A crucial parameter determining the clinical relevance of a CEP is the prediction of TL. It could be assumed that not meeting CEPs would lead to a higher rate of TL. In the Austrian cohort, CEP1 failure was identified as a predictor of future PTL over a mean period of 10.7 years of SPC (Bertl et al. 2022). In a larger cohort, only failure to achieve CEP2 was significantly correlated with TL. No significant associations were found between CEP1 and TL. Additionally, only a small percentage of teeth were lost for any reason during SPC in adherent patients: a total of 3.1% of teeth were lost in one‐third of the subjects included, based on 5‐year data (Rattu et al. 2023). In the present study, achievement of CEP2 and CEP3 was significantly associated with no PTL, compared to CEP4 and noCEP. Achievement of CEP1 had a very low OR for PTL.

Success of PRA‐driven SPC with high patient adherence was also observed in this study. At T2, only 12 patients (noCEP, 9.4%) exceeded the criteria of the department‐specific threshold value above which a case is considered to require re‐treatment (CEP4). While it is favourable to achieve a stricter CEP, this study failed to show significant differences in terms of clinical parameter evolution from T1 to T2 between CEP1, CEP2 and CEP3. However, this lack of difference may hide clinically relevant factors. CEP1 does not allow PPD = 4 mm with BOP and PPD > 4 mm. This means that for a CEP1 patient, there is no need for repeated subgingival instrumentation (SI) during SPC (Sanz et al. 2020). With CEP2, there may be PPD ≥ 4 mm and < 6 mm (Sanz et al. 2020), and with CEP3, there may be up to 4 sites with PPD ≥ 5 mm and variable numbers of sites/teeth with PPD = 4 mm and BOP (Feres et al. 2020). In these patients, these sites require SI (Sanz et al. 2020). As a consequence, CEP1 is preferable over CEP2 and CEP3, because clinical parameters (i.e., number of sites with PPD > 5 mm) in a patient achieving CEP1 plausibly will require less effort to be kept stable.

This analysis confirms the observation that long‐term (10 years) prognosis is difficult to determine based on early diagnosis and clinical parameters (McGuire and Nunn 1996). This is true for several reasons. First, CEPs do not account for systemic risk modifiers, such as smoking or diabetes. Second, various patient‐related factors may change over time: patients may start or stop smoking, and they may suffer from depression or other diseases that were not considered at T1. However, it still makes sense to define CEPs that provide an estimate for future stability. Meanwhile, SPC must be adjusted over time to tackle unexpected challenges.

What are the clinical consequences of success or failure in achieving any of the investigated CEPs? First, the investigated CEPs consist of routinely assessed clinical parameters (i.e., PPD and in part BOP) that are readily available after APT. They provide a rough estimation of whether the respective patient may be transferred into SPC or not. However, 59% of all patients included in this study did not achieve CEP1 to CEP3 after APT (T1). Does this mean that APT should have been continued in more than one‐third of all patients until CEP1, 2 or 3 was achieved? The investigated CEPs serve as orientation. Achieving one of these CEPs after APT suggests a high likelihood of maintaining stability during SPC. If APT failed to achieve CEP1, 2 or 3, it is a matter of the respective patient and clinician to discuss and decide how to proceed (participative decision‐making). Possible options include attempting another cycle of APT to achieve at least CEP3, allowing more time for tissues to heal or accepting the risk of some more instability and proceeding to SPC that may be scheduled more frequently to manage residual pockets (Ramseier et al. 2019).

At T2, 63% of all patients achieved CEP1, 2 or 3 demonstrating at least some positive effect of 10 years of SPC. However, treatment during SPC must always be adjusted according to changing conditions, such as adherence and smoking status.

This study has limitations, including its retrospective design, which may have introduced selection bias. Another limitation is the classification of CEPs as mutually exclusive. While this structure was necessary to facilitate comparison, the true overlap of CEP is likely to dilute differences in long‐term stability. Thus, we did not find differences in long‐term periodontal stability between mutually exclusively assigned CEPs. The overlap of CEPs means differences are even less likely than we report. However, models to predict achievement of CEPs at T1 by parameters at T0 and at T2 by parameters at T1 were calculated for actual CEPs that encompass all qualifying patients. The additional sensitivity analyses revealed that the results of the original analysis were only partially replicable with this model (Tables S3 and S4). This indicates that our results should be interpreted with caution.

In conclusion, the findings of this retrospective cohort study are largely consistent with those of recent studies investigating CEP achievement. Only a minority of patients achieved CEP1, and there was a lack of significant difference in long‐term (T1–T2) stability between patients achieving CEP1, CEP2 and CEP3 at T1. Thus, CEP2 (Sanz et al. 2020) and CEP3 (Feres et al. 2020) seem to be as effective in providing long‐term stability as the supposedly ‘ideal’ CEP1 (Chapple et al. 2018). However, because they allow pockets of 4 mm with bleeding and some sites with 5 mm, prolonged stability with CEP2 and CEP3 may require frequently repeated SI and APT.

## Author Contributions

All authors contributed substantially to the interpretation of the data for the work, they contributed to the drafting and critical revision of the manuscript, gave their final approval of the version to be published and agreed to be accountable for all aspects of the work. Additionally, H.P. and P.E. conceived the ideas for the concept and design of the study; M.S. and M.B. collected the data; M.S. and M.B. complied with methodical approaches; I.D. analysed the data; H.P. secured funding and managed the group; P.E. led the writing.

## Ethics Statement

All procedures performed in the study were approved by the Institutional Review Board for Human Studies of the Medical Faculty of Johann Wolfgang Goethe‐University Frankfurt am Main (approval number: 61/15), conducted in accordance with the 1975 Declaration of Helsinki as revised in 2013.

## Conflicts of Interest

The authors declare no conflicts of interest.

## Supporting information


**Data S1.** Supporting information.

## Data Availability

The data that support the findings of this study are available from the corresponding author upon reasonable request.

## References

[jcpe14179-bib-0001] Bertl, K. , N. Pandis , N. Stopfer , H. Haririan , C. Bruckmann , and A. Stavropoulos . 2022. “The Impact of a ‘Successfully Treated Stable Periodontitis Patient Status’ on Patient‐Related Outcome Parameters During Long‐Term Supportive Periodontal Care.” Journal of Clinical Periodontology 49: 101–110. 10.1111/jcpe.13582. 34866227

[jcpe14179-bib-0002] Chambrone, L. A. , and L. Chambrone . 2006. “Tooth Loss in Well‐Maintained Patients With Chronic Periodontitis During Long‐Term Supportive Therapy in Brazil.” Journal of Clinical Periodontology 33: 759–764. 10.1111/j.1600-051X.2006.00972.x. 16899027

[jcpe14179-bib-0003] Chapple, I. L. C. , B. L. Mealey , T. E. Van Dyke , et al. 2018. “Periodontal Health and Gingival Diseases and Conditions on an Intact and a Reduced Periodontium: Consensus Report of Workgroup 1 of the 2017 World Workshop on the Classification of Periodontal and Peri‐Implant Diseases and Conditions.” Journal of Clinical Periodontology 45: S68–S77. 10.1111/jcpe.12940. 29926499

[jcpe14179-bib-0004] Cortellini, P. , M. S. Tonetti , N. P. Lang , et al. 2001. “The Simplified Papilla Preservation Flap in the Regenerative Treatment of Deep Intrabony Defects: Clinical Outcomes and Postoperative Morbidity.” Journal of Periodontology 72: 1702–1712. 10.1902/jop.2001.72.12.1702. 11811506

[jcpe14179-bib-0005] Eickholz, P. , J. Kaltschmitt , J. Berbig , P. Reitmeir , and B. Pretzl . 2008. “Tooth Loss After Active Periodontal Therapy. 1: Patient‐Related Factors for Risk, Prognosis, and Quality of Outcome.” Journal of Clinical Periodontology 35: 165–174. 10.1111/j.1600-051X.2007.01184.x. 18199150

[jcpe14179-bib-0006] Eickholz, P. , Y. Siegelin , S. Scharf , et al. 2013. “Non‐Surgical Periodontal Therapy Decreases Serum Elastase Levels in Aggressive but Not in Chronic Periodontitis.” Journal of Clinical Periodontology 40: 327–333. 10.1111/jcpe.12076. 23432024

[jcpe14179-bib-0007] Faggion, C. M., Jr. , G. Petersilka , D. E. Lange , J. Gerss , and T. F. Flemmig . 2007. “Prognostic Model for Tooth Survival in Patients Treated for Periodontitis.” Journal of Clinical Periodontology 34: 226–231. 10.1111/j.1600-051X.2006.01045.x. 17257157

[jcpe14179-bib-0008] Fardal, O. , and G. J. Linden . 2005. “Re‐Treatment Profiles During Long‐Term Maintenance Therapy in a Periodontal Practice in Norway.” Journal of Clinical Periodontology 32: 744–749. 10.1111/j.1600-051X.2005.00778.x. 15966881

[jcpe14179-bib-0009] Feres, M. , B. Retamal‐Valdes , M. Faveri , et al. 2020. “Proposal of a Clinical Endpoint for Periodontal Trials: The Treat‐To‐Target Approach.” Journal of the International Academy of Periodontology 22: 41–53. 32224549

[jcpe14179-bib-0010] Herrera, D. , M. Sanz , M. Kebschull , et al. 2022. “Treatment of Stage IV Periodontitis: The EFP S3 Level Clinical Practice Guideline.” Journal of Clinical Periodontology 49: 4–71. 10.1111/jcpe.13639. 35688447

[jcpe14179-bib-0011] Hujoel, P. P. 2004. “Endpoints in Periodontal Trials: The Need for an Evidence‐Based Research Approach.” Periodontology 2000 36: 196–204. 10.1111/j.1600-0757.2004.03681.x. 15330950

[jcpe14179-bib-0012] Loos, B. G. , and I. Needleman . 2020. “Endpoints of Active Periodontal Therapy.” Journal of Clinical Periodontology 47, no. Suppl 22: 61–71. 10.1111/jcpe.13253. 31912527 PMC7670400

[jcpe14179-bib-0013] McGuire, M. K. , and M. E. Nunn . 1996. “Prognosis Versus Actual Outcome. III. The Effectiveness of Clinical Parameters in Accurately Predicting Tooth Survival.” Journal of Periodontology 67: 666–674. 10.1902/jop.1996.67.7.666. 8832477

[jcpe14179-bib-0014] Müller, S. , P. Eickholz , P. Reitmeir , and T. Eger . 2013. “Long‐Term Tooth Loss in Periodontally Compromised but Treated Patients According to the Type of Prosthodontic Treatment. A Retrospective Study.” Journal of Oral Rehabilitation 40: 358–367. 10.1111/joor.12035. 23362962

[jcpe14179-bib-0015] Nyman, S. , J. Lindhe , and B. Rosling . 1977. “Periodontal Surgery in Plaque‐Infected Dentitions.” Journal of Clinical Periodontology 4: 240–249. 10.1111/j.1600-051x.1977.tb01896.x. 340476

[jcpe14179-bib-0016] Papapanou, P. N. , M. Sanz , N. Buduneli , et al. 2018. “Periodontitis: Consensus Report of Workgroup 2 of the 2017 World Workshop on the Classification of Periodontal and Peri‐Implant Diseases and Conditions.” Journal of Clinical Periodontology 45: S162–S170. 10.1111/jcpe.12946. 29926490

[jcpe14179-bib-0017] Petsos, H. , T. Ramich , K. Nickles , et al. 2021. “Tooth Loss in Periodontally Compromised Patients: Retrospective Long‐Term Results 10 Years After Active Periodontal Therapy – Tooth‐Related Outcomes.” Journal of Periodontology 92: 1761–1775. 10.1002/JPER.21-0056. 33748997

[jcpe14179-bib-0018] Petsos, H. , B. Schacher , T. Ramich , et al. 2020. “Retrospectively Analysed Tooth Loss in Periodontally Compromised Patients: Long‐Term Results 10 Years After Active Periodontal Therapy—Patient‐Related Outcomes.” Journal of Periodontal Research 55: 946–958. 10.1111/jre.12786. 33145760

[jcpe14179-bib-0019] Quirynen, M. , C. M. L. Bollen , B. N. A. Vandekerckhove , C. Dekeyser , W. Papaioannou , and H. Eyssen . 1995. “Full‐ Vs. Partial‐Mouth Disinfection in the Treatment of Periodontal Infections: Short‐Term Clinical and Microbiological Observations.” Journal of Dental Research 74: 1459–1467. 10.1177/00220345950740080501. 7560400

[jcpe14179-bib-0020] Ramseier, C. A. , M. Nydegger , C. Walter , et al. 2019. “Time Between Recall Visits and Residual Probing Depths Predict Long‐Term Stability in Patients Enrolled in Supportive Periodontal Therapy.” Journal of Clinical Periodontology 46: 218–230. 10.1111/jcpe.13041. 30499586

[jcpe14179-bib-0021] Rattu, V. , D. Raindi , G. Antonoglou , and L. Nibali . 2023. “Prevalence of Stable and Successfully Treated Periodontitis Subjects and Incidence of Subsequent Tooth Loss Within Supportive Periodontal Care: A Systematic Review With Meta‐Analyses.” Journal of Clinical Periodontology 50: 1371–1389. 10.1111/jcpe.13835. 37402624

[jcpe14179-bib-0022] Sanz, M. , D. Herrera , M. Kebschull , et al. 2020. “Treatment of Stage I‐III Periodontitis‐The EFP S3 Level Clinical Practice Guideline.” Journal of Clinical Periodontology 47, no. Suppl 22: 4–60. 10.1111/jcpe.13290. 32383274 PMC7891343

[jcpe14179-bib-0023] Tonetti, M. S. , H. Greenwell , and K. S. Kornman . 2018. “Staging and Grading of Periodontitis: Framework and Proposal of a New Classification and Case Definition.” Journal of Periodontology 89: S159–S172. 10.1002/JPER.18-0006. 29926952

