# Supplementary material for: Clinical Treatment Endpoints After Active Periodontal Treatment and 10 Years of Supportive Periodontal Care: A Retrospective Cohort Study
Source: J Clin Periodontol. 2025 Jul 8;52(10):1386–97. doi: 10.1111/jcpe.14179 (PMC12420075; doi:10.1111/jcpe.14179)
Supplement: Supplementary file 1 — Data S1. Supporting information. [file JCPE-52-1386-s001.docx]

Supplementary Text

Materials and methods

**Protocol of periodontal treatment**

All patients received oral hygiene instructions and professional prophylaxes until the modified Plaque Control Record (PCR) (O'Leary et al., 1972) was ≤ 50%. SI was performed over two visits on two consecutive days. On the first day, the right side (1^st^ and 4^th^ quadrants) was treated, and on the following day the left side (2^nd^ and 3^rd^ quadrants). Immediately after receiving local anesthesia (UDS, Sanofi-Aventis Deutschland GmbH, Frankfurt/Main, Germany) each patient brushed the back of the tongue for 60 seconds with 1% chlorhexidine (CHX) gel (Chlorhexamed 1% Gel, GlaxoSmithKline, München, Germany) and rinsed twice, for 60 seconds each, with 10 ml of 0.12% CHX solution (ParoEx, John O. Butler, Kriftel, Germany). For the last 10 seconds, patients were advised to gargle. All teeth exhibiting PPD ≥3.5 mm were subgingivally debrided using sonic scalers (Sonicsys, KaVo, Biberach, Germany) and hand instruments. Immediately after instrumentation, 1% CHX gel was applied to all debrided pockets three times within 10 minutes. In aggressive or severe chronic periodontitis (stage III and IV, grade B and C) cases subgingival plaque was analysed using commercially available tests. If *Aggregatibacter actinomycetemcomitans* was detected, 500 mg amoxicillin and 400 mg metronidazole were prescribed (three times daily for seven days). In case of sensitivity to penicillin, 250 mg ciprofloxacin and 500 mg metronidazole were prescribed (twice daily for seven days) (Beikler et al. 2003, Griffith et al. 2011, Feres et al. 2012). Six days after debridement, subgingival application of 1% CHX gel was repeated. For all patients, oral home care was instructed for 14 days after the start of SRP, including rinsing twice daily for 60 seconds with 10 ml 0.12% CHX solution (ParoEx), before brushing the teeth and back of the tongue with 1% CHX gel. Six and 12 weeks after subgingival debridement, all patients received oral hygiene instructions and professional prophylaxis.

If sites with PPD ≥6 mm following SI were present, periodontal surgery was recommended. Inclusion of patients only after October 2004/April 2005 ensured consistent application of a standardized and uniform treatment protocol (Eickholz et al., 2013) during APT and SPC; this follows the change of the head of the department and the introduction of this treatment concept in October 2004.

**Inclusion criteria**

Inclusion criteria for participation in this retrospective cohort study were as follows:

1. Treatment according to the previously described concept (Eickholz et al., 2013)
2. Complete periodontal examination [PPD, clinical attachment levels (CAL) and BOP at six sites per tooth, furcation involvement (Hamp et al., 1975) at all furcation sites of multi-rooted teeth] performed before start of therapy (baseline, T0), after completion of APT -including non-surgical or surgical therapy- at the start of SPC (T1) and after long-term SPC (T2)
3. Age ≥18 years at the time of re-examination (T2)
4. T1-T2=120±12 months
5. Written informed consent

**Supportive periodontal care**

1. Assessment of modified Gingival Bleeding Index (GBI (Ainamo and Bay, 1975)) and modified PCR (O'Leary et al., 1972) at six sites per tooth (Petsos et al., 2020)
2. Re-instruction and re-motivation for an effective individual plaque control
3. Professional mechanical plaque removal using hand instruments and polishing with rotating rubber cups and polishing paste (SuperPolish; Kerr GmbH)
4. Application of fluoride gel (Elmex Gelée; GABA Schweiz AG) (Axelsson and Lindhe, 1975)
5. Once or twice a year, a general dental examination and a complete periodontal status including PPD, CAL, BOP, furcation involvement, and tooth mobility were recorded. Sensitivity testing was performed at least once per year. At sites with PPD = 4 mm + BOP or PPD ≥5 mm, SI was performed and 1% chlorhexidine digluconate gel (Chlorhexamed 1% gel; GlaxoSmithKline GmbH) was subgingivally instilled.

Patients classed as low or medium risk in Periodontal Risk Assessment (PRA) according to Lang and Tonetti (2003) were scheduled for one or two annual visits, respectively. Patients with high risk were scheduled for four annual visits and received complete SPC at six-month intervals. In between, SPC without dental and periodontal charting was provided. If a patient exhibited >5 teeth each with PPD ≥5 mm (CEP4) two years after the end of APT, periodontal re-treatment was recommended; individual factors such as the patient's age, timing of re-evaluation, and systemic diseases were taken into account (Petsos et al., 2020). To classify adherence (adherent or non-adherent), SPC interval recommendations were compared with the intervals documented in the patient’s file. If a patient exceeded the interval determined during SPC once by more than 100%, they were considered to be non-adherent [for example, the recommended interval was 6 months, but the patient did not return to SPT until after 13 months (Eickholz et al., 2008)].

**10 year re-examination (T2)**

1. Self-reported smoking status [non-smokers (never smoked), former smokers (stopped smoking ≥5 years ago), and active smokers (stopped smoking <5 years ago or currently smoking)] (Lang and Tonetti, 2003)
2. Medical history
3. Dental status
4. Modified GBI (Ainamo and Bay, 1975) and modified PCR (O'Leary et al., 1972)
5. PPD and CAL to the nearest 1.0 mm with a manual, millimetre-scaled rigid periodontal probe (PCPUNC 15, Hu-Friedy) at six sites per tooth; BOP and suppuration were recorded 30 seconds after probing
6. Furcation involvement (Eickholz and Walter, 2018, Hamp et al., 1975) at all multi-rooted teeth with a Nabers probe (PQ2N, Hu-Friedy)
7. Adherence (adherent/non-adherent), defined as described above
8. Patients who experienced TL were asked about the reasons, particularly if teeth were removed outside of the Center of Dentistry and Oral Medicine (Carolinum). For patients whose teeth were removed in the Center or the authors’ department, reasons for extraction -such as periodontal diseases [combination of progressive CAL loss, furcation involvement II/III (Hamp et al., 1975) and/or tooth mobility II/III (Nyman et al., 1975)], caries or secondary caries diseases (carious lesions that could not be restored, endodontic complications that could not be managed by a revision), orthodontics (lack of space, balancing extractions), prosthetic considerations (unusable as an abutment tooth), or trauma (longitudinal, untreatable fractures)- were verified from the patient file (Petsos et al., 2020, Petsos et al., 2021). PTL was assessed.

Due to inconsistent and sometimes insufficient documentation of extraction justifications over the past 10 years, the most recent clinical and radiological findings before extraction were used, unless explicitly documented, to determine if there were valid periodontal reasons or other mentioned factors influencing the decision for extraction.

All examiners were experienced periodontists who had completed a minimum of three years of postgraduate training. The inter-individual calibration of all participating dentists for PPD and CAL has already been described previously (Petsos et al., 2020).

**Statistical analysis**

Significant predictors identified by the univariate regressions based on the parameters recorded at the previous time point (T0 or T1) were included in the corresponding multiple regression: gender, age, smoking, diabetes, stage, grade, antibiotics intake, surgery, number of teeth, full-mouth mean PPD, number of sites with PPD >5 mm, number of sites with PPD >6 mm, full-mouth mean CAL, periodontal inflamed surface area (PISA), number of mobile teeth (class II and class III), and number of teeth with furcation involvement (class II and class III). The regression model for T1 CEPs included the following T0 predictors: diabetes, number of teeth, PISA, and the number of sites with PPD >5 mm. The regression model for T2 CEPs included the following T1 predictors: age, mean CAL, surgery, number of sites with PPD >6 mm, smoking, and PISA. The significance level in all analyses, including the multivariate analysis, was set at 5%.

To correct for performing several multiple regressions, we did a sensitivity analysis performing for each time point an ordinal regression (R-package “MASS”) for the ordinal dependent variable with value equal to the number of not achieved actual CEPs (i.e., equal to 1 if actual CEP1=1, equal to 2 if actual CEP2=1 and so on and 5 if noCEP=1). As predictors for each time point we considered the variables that were kept in the multiple regressions in the original analysis. Rank-based independence tests, stratified by the confounding variables “smoking” and “diabetes”, were performed for each variable measured at T2 to analyze the influence of the CEP achieved at T1 on stability (R-package “coin”, see https://cran.r-project.org/web/packages/coin/vignettes/Implementation.pdf for references to the independence tests).

Supplementary Figures


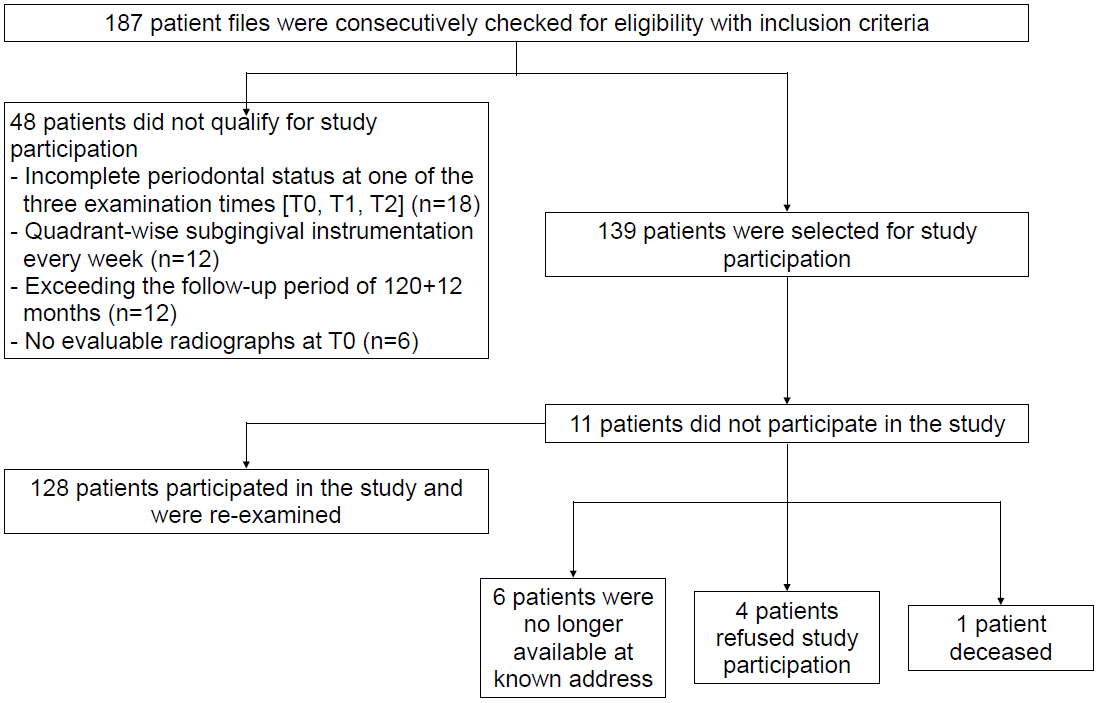


**Figure 1 Suppl. CONSORT flow diagram (Petsos et al., 2021)**


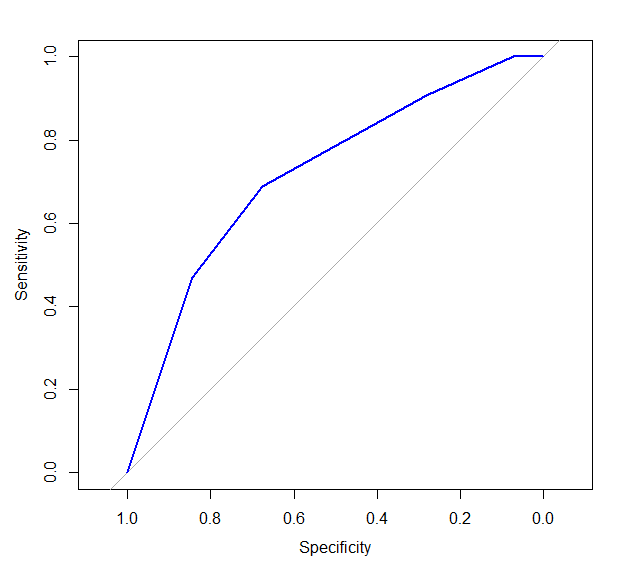


Figure 2 Suppl. ROC curve for the prediction of PTL_SCP based on the best achieved CEP at T1

Supplementary Tables

| **Table 1 Suppl. Univariate logistic regressions for parameters at baseline (T0) predicting clinical endpoints (CEP) at T1** | | | | |
| --- | --- | --- | --- | --- |
| CEP  Predictor (T0) | CEP1 | Actual CEP2 (CEP1+CEP2) | Actual CEP3 (CEP1+CEP2+CEP3) | Actual CEP4 (CEP1+CEP2+CEP3+CEP4) |
|  | p-value | p-value | p-value | p-value |
| Gender (reference: female) | 0.667 | 0.462 | 0.975 | 0.251 |
| Age | 0.132 | 0.278 | 0.77 | 0.435 |
| Smoking (reference: non-smoker) | 0.674 | 0.907 | 0.199 | 0.061 |
| Diabetes (reference: non-diabetic) | **0.007** | 0.384 | 0.343 | 0.989 |
| Stage (reference: stage III) | 0.662 | 0.526 | 0.54 | 0.526 |
| Grade (reference: grade B) | 0.730 | 0.67 | 0.841 | 0.67 |
| Antibiotics (reference: no antibiotic intake) | 0.995 | 0.991 | 0.285 | **0.011** |
| Surgery (reference: no surgery) | 0.293 | 0.062 | 0.121 | 0.15 |
| Number of teeth | **0.029** | 0.926 | 0.329 | 0.445 |
| PPD | 0.119 | **0.006** | **0.021** | **0.002** |
| PPD >5 mm (reference: PPD ≤4 mm) | 0.054 | **0.004** | **0.009** | **<0.001** |
| PPD >6 mm (reference: PPD ≤5 mm) | 0.054 | **0.002** | **0.009** | **0.003** |
| CAL | 0.812 | 0.109 | 0.108 | **0.02** |
| PISA | **0.044** | 0.099 | **0.027** | **0.006** |
| Mobility (0, I/II, III) | 0.065 | 0.07 | **0.025** | 0.105 |
| Furcation (0, I/II, III) | 0.992 | 0.866 | 0.546 | 0.465 |

SD = standard deviation, TL = tooth loss, PTL = periodontal tooth loss, PPD = periodontal probing depth, CAL = clinical attachment level,

PISA = periodontally inflamed surface area

| **Table 2 Suppl. Univariate logistic regressions for parameters after APT (T1) predicting clinical endpoints (CEP) at T2** | | | | |
| --- | --- | --- | --- | --- |
| CEP  Predictor (T1) | CEP1 | Combined CEP2 (CEP1+CEP2) | Combined CEP3 (CEP1+CEP2+CEP3) | Combined CEP4 (CEP1+CEP2+CEP3+CEP4) |
|  | p-value | p-value | p-value | p-value |
| Gender (reference: female) | 0.969 | 0.072 | 0.435 | 0.955 |
| Age | **0.015** | 0.334 | 0.217 | 0.205 |
| Smoking (reference: non-smoker) | 0.995 | 0.079 | **0.041** | **0.034** |
| Diabetes (reference: non-diabetic) | 0.993 | 0.52 | 0.877 | 0.993 |
| Stage (reference: stage III) | 0.121 | 0.461 | 0.841 | 0.89 |
| Grade (reference: grade B) | 0.142 | **0.017** | 0.108 | 0.382 |
| Antibiotics (reference: no antibiotic intake) | 0.995 | 0.469 | 0.598 | 0.307 |
| Surgery (reference: no surgery) | 0.147 | 0.291 | 0.122 | 0.623 |
| Teeth | 0.091 | 0.988 | 0.889 | 0.267 |
| TL SPC (reference: no TL) | 0.871 | 0.840 | 0.818 | 0.564 |
| PTL SPC (reference: no PTL) | 0.490 | 0.618 | 0.136 | 0.549 |
| PPD | 0.060 | 0.235 | 0.072 | 0.387 |
| PPD >5 mm (reference: PPD ≤4 mm) | 0.077 | 0.084 | **0.005** | 0.366 |
| PPD >6 mm (reference: PPD ≤5 mm) | 0.148 | 0.294 | **0.001** | 0.606 |
| CAL | **0.026** | 0.619 | 0.551 | 0.506 |
| PISA | 0.144 | 0.076 | 0.088 | 0.043 |
| Mobility (0, I/II, III) | 0.994 | 0.412 | 0.369 | 0.754 |
| Furcation (0, I/II, III) | 0.753 | 0.052 | 0.094 | 0.181 |

APT = active periodontal treatment, SD = standard deviation, TL = tooth loss, PTL = periodontal tooth loss, PPD = periodontal probing depth, CAL = clinical attachment level,

PISA = periodontally inflamed surface area

| **Tab. 3 Supplement. Sensitivity analysis for prediction of CEPs at T1 by parameters at T0** | | | |
| --- | --- | --- | --- |
| Predictor T0 | Regression coefficient | Standard error | p-value |
| Diabetes (reference: non-diabetic) | -1.403 | 1.053 | 0.183 |
| Number of teeth | 0.074 | 0.055 | 0.176 |
| PISA | 0.004 | 0.001 | 0.446 |
| PPD >5 mm (reference: PPD ≤4 mm) | 1.049 | 0.148 | **<0.001** |
| PPD >6 mm (reference: PPD ≤5 mm) | -0.234 | 0.167 | 0.163 |
| Mobility (0. I/II. III) | -0.184 | 0.421 | 0.662 |

PISA = periodontally inflamed surface area

statistic: multiple ordinal regression

| **Tab. 4 Supplement. Sensitivity analysis for prediction of CEPs at T2 by parameters at T1** | | | |
| --- | --- | --- | --- |
| Predictor T1 | Regression coefficient | Standard error | p-value |
| Age | 0.022 | 0.017 | 0.215 |
| CAL | -0.208 | 0.227 | 0.359 |
| grade | 0.911 | 0.346 | 0.008 |
| Smoking (reference: non-smoker) | 0.684 | 0.254 | **0.007** |
| PPD >5 mm (reference: PPD ≤4 mm) | 0.054 | 0.057 | 0.345 |
| PPD >6 mm (reference: PPD ≤5 mm) | 0.072 | 0.109 | 0.505 |

CAL = clinical attachment level

statistic: multiple ordinal regression

| **Tab. 5 Supplement. Measures for the prediction of PTL_SPC based on the best achieved CEP at T1** | | | | |
| --- | --- | --- | --- | --- |
| Threshold at T1 | Sensitivity | Specificity | PPV | NPV |
| CEP1 | 1 | 0 | 0.25 | - |
| CEP2 | 1 | 0.073 | 0.264 | 1 |
| CEP3 | 0.906 | 0.281 | 0.296 | 0.9 |
| CEP4^§^ | 0.688 | 0.677 | 0.415 | 0.867 |
| NoCEP | 0.469 | 0.844 | 0.5 | 0.827 |

* Prediction rule: predicted PTL_SPC=1 if best achieved CEP at T1 >=threshold

§ Best cutoff based on Youden’s Index
